# The Local Anesthetic Bupivacaine Inhibits the Progression of Non-Small Cell Lung Cancer by Inducing Autophagy Through Akt/mTOR Signaling

**DOI:** 10.3389/fonc.2021.616445

**Published:** 2021-03-11

**Authors:** Jian-Hua Gu, Cui-Cui Liu, Jin-Lan Xie, Bin Ma, Shao-Min Cui, Guang-Zhu Yang, Shun-Cheng He

**Affiliations:** Department of Anesthesia, Jinan People’s Hospital, Jinan, China

**Keywords:** NSCLC, progression, bupivacaine, autophagy, Akt/mTOR signaling

## Abstract

Non-small cell lung cancer (NSCLC) is a prevalent malignancy with high mortality and poor prognosis. Bupivacaine serves as a widely used local anesthetic and presents potential anti-tumor activity. Nevertheless, the function of bupivacaine in the NSCLC development remains elusive. Here, we tried to investigate the impact of bupivacaine on the NSCLC progression. Significantly, we revealed that bupivacaine was able to reduce the proliferation and induce the apoptosis of NSCLC cells. Bupivacaine could attenuate the invasion and migration in the cells. Mechanically, the treatment of bupivacaine increased the expression ratio of light chain 3B-II (LC3B-II)/LC3B-I and the expression of Beclin-1 in the NSCLC cells. Meanwhile, the expression of the autophagic adaptor protein p62 was decreased by bupivacaine treatment in the cells. The treatment of bupivacaine attenuated the phosphorylation of AKT and mTOR in the NSCLC cells. The AKT activator SC79 and autophagy inhibitor 3-methyladenine (3-MA) reversed the bupivacaine-inhibited phosphorylation of AKT and mTOR and bupivacaine-induced autophagy, as well as the bupivacaine-attenuated NSCLC progression in the cells. Bupivacaine could inhibit the tumor growth *in vivo*. In conclusion, we discovered that the local anesthetic bupivacaine inhibited the progression of NSCLC by inducing autophagy through Akt/mTOR signaling. Our finding provides new insights into the mechanism by which bupivacaine attenuates the development of NSCLC. Bupivacaine may serve as a potential anti-tumor candidate for the therapeutic strategy of NSCLC.

## Introduction

Lung cancer serves as the most common malignancy and is the principal cause of tumor-related mortality globally, according to the latest annual statistics report of global cancer (1). Non-small cell lung cancer (NSCLC) accounts for about 83% of primary lung cancer (2). At present, surgical resection is the only radical treatment for NSCLC, while most patients with NSCLC are diagnosed at advanced stages, which is not appropriate for surgical operation (3). Therefore, early diagnosis and treatment are pivotal to improve treatment outcomes of NSCLC (4). Unfortunately, the 5-year survival for NSCLC cases remains poor, and the recurrence rate in the cases is high because of drug-resistance or tumor metastasis (5). Hence, the development of more practical drug candidates and therapeutic strategy is urgently needed (6). However, the advancement in this research field is still limited.

Bupivacaine serves as a local amide-linked anesthetic and is usually employed to relieve pain in patients with cancers during or after neoplasm removal operation, such as gastric neoplasia endoscopic dissection (7). The mechanism of bupivacaine’s action as anesthesia is the interference of voltage-gated sodium-channel blockade, inhibiting depolarization in nerve cells (8). Significantly, several investigations based on preclinical models illustrate that bupivacaine promotes cancer cell death and inhibits cancer progression at a range of specific concentrations in many cancer models, including prostate and ovarian cancer (9, 10). However, the effect of bupivacaine on NSCLC progression remains unclear.

Autophagy is an intracellular process that cellular contents, such as dysfunctional organelles and large protein groups, are transported to lysosomes for degradation and reuse (11). By the degradation and recycling of dysfunctional or unnecessary intracellular ingredients, autophagy sustains cellular homeostasis and limits cellular damage under multiple stresses (12). Autophagy works as a double-role brand in cancer development (13). In several cases, autophagy induces cancer cell survival by recovering intracellular ingredients and increasing energy generation to reach cancer cells’ high metabolic requirements. In other cases, autophagy decreases cell imbalance and injury to inhibit tumorigenesis (14). It has been identified that the activation of autophagy is involved in the anti-tumor processes in NSCLC (15). Meanwhile, as a critical pathway and potential drug target in cancer development, Akt/mTOR signaling stimulates cell proliferation at various stages of the cell cycle (16). Akt/mTOR signaling activation ultimately causes autophagy repression in cancer cells and increases NSCLC development, rendering resistance of NSCLC cells to various cancer therapies and leading to a poor prognosis (16, 17). However, the effect of bupivacaine on autophagy and Akt/mTOR signaling in the development of NSCLC is still elusive.

In the present study, we focused on the investigation of the impact and the underlying mechanism of bupivacaine on the development of NSCLC. We identified a novel anti-tumor function of bupivacaine in NSCLC progression by activating autophagy through inhibiting Akt/mTOR signaling.

## Materials and Methods

### Cell Culture

The A549 and H1299 cells were obtained in American Type Tissue Culture Collection. The cells were cultured in the medium of RPMI-1640 (Gibco, USA) containing 0.1 mg/ml of streptomycin (Gibco, USA), 100 units/ml of penicillin (Gibco, USA), and 10% fetal bovine serum (Gibco, USA), at a condition of 37°C with 5% CO_2_. Bupivacaine (Sigma, USA) was dissolved in DMSO and diluted by physiological saline. The SC79 and 3-methyladenine (3-MA) were purchased from Sigma-Aldrich (St. Louis, MO, USA).

### MTT Assays

The cell viability was measured by MTT assays. Briefly, about 2 × 10^4^ cells were put into 96 wells and cultured for 12 h. After indicated treatment, the cells were added with the MTT solution (10 μL, 5 mg/ml) and cultured for an extra 4 h. Discarded medium, and 150 μL DMSO was used to treat the wells. An ELISA browser was applied to analyze the absorbance at 570 nm (Bio-Tek EL 800, USA).

### Colony Formation Assays

About 1 × 10^3^ A549 and H1299 cells were layered in 6 wells and incubated in DMEM at 37 °C. After two weeks, cells were cleaned with PBS Buffer, made in methanol about 30 min, and dyed with crystal violet dye at the dose of 1%, after which the number of colonies was calculated.

### BrdU Incorporation Assays

The cell proliferation was assessed by the BrdU incorporation assays. About 2 × 10^3^ cells were put into 96 wells and cultured for 12 h. Then the cells were used for the transfection or treatment. After 0, 24, 48, and 72 h, cell proliferation was measured by BrdU Cell Proliferation Assay Kit (Yilaisa, China).

### Transwell Assays

Transwell assays analyzed the impacts of bupivacaine on cell migration and invasion of NSCLC by applying a Transwell plate (Corning, USA) according to the manufacturer’s guidance. Briefly, the upper chambers were plated with around 1 × 10^5^ cells. Then solidified using paraformaldehyde (4%) and dyed using crystal violet. Invaded and migrated cells were recorded and calculated.

### Wound Healing Assay

A549 and H1299 cells were put in the 24-well plate at 3 × 10^5^/well and cultured overnight to reach a full confluent as a monolayer. A 20μl pipette tip was applied to slowly cut a straight line across the well. Then the well was washed by PBS 3 times and changed with the serum-free medium and continued to culture. The wound healing percentage was calculated.

### Analysis of Cell Apoptosis

About 2 × 10^5^ cells were plated on 6-well dishes. Cell apoptosis was assessed by employing the Annexin V-FITC Apoptosis Detection Kit (CST, USA) using the manufacture’s instruction. Briefly, about 2 × 10^6^ collected and washed cells collected by binding buffer and were dyed at 25 °C, followed by the flow cytometry analysis.

### Western Blot Analysis

Total proteins were obtained from the mice tissues or cells with RIPA buffer (CST, USA). Protein concentrations were analyzed by applying the BCA Protein Quantification Kit (Abbkine, USA). Same concentration of protein was divided by SDS-PAGE (12% polyacrylamide gels), transferred to PVDF membranes (Millipore, USA) in the subsequent step. The membranes were hindered with 5% milk and hatched overnight at 4°C with the primary antibodies for Bcl-2 (Abcam, USA), Bax (Abcam, USA), cleaved caspase3 (Abcam, USA), LC3B (Abcam, USA), p62 (Abcam, USA), Beclin-1 (Abcam, USA), AKT (Abcam, USA), mTOR (Abcam, USA), p-AKT (Abcam, USA), p-mTOR (Abcam, USA), and β-actin (Abcam, USA), in which β-actin served as the control. Then, the corresponding second antibodies (Abcam, USA) were used for hatching the membranes 1 h at room temperature, followed by the visualization by using an Odyssey CLx Infrared Imaging System.

### Analysis of Tumorigenicity in Nude Mice

The effect of bupivacaine on tumor growth of NSCLC *in vivo* was analyzed in nude mice of Balb/c (male, 4-week-old) (n = 5). About 1 × 10^7^ cells A549 cells were subcutaneously injected into the mice. The mice were treated with bupivacaine (40 μmol/kg) or equal volume saline. After 7 days of injection, we measured tumor growth every 7 days. We sacrificed the mice after 28 days of injection, and tumors were scaled. Tumor volume (V) was observed by estimating the length and width with calipers and measured with the method × 0.5. Animal care and method procedure were authorized by the Animal Ethics Committee of Jinan People’s Hospital, and were carried out in accordance with the National Institutes of Health guide for the care and use of Laboratory animals.

### Statistical Analysis

Data were expressed as mean ± SD, and the statistical analysis was conducted using GraphPad prism 7. The unpaired Student’s t-test was used to compare two groups, and the one-way ANOVA was used to compare among multiple groups. P < 0.05 was considered as statistically significant.

## Results

### Bupivacaine Inhibits Proliferation and Promotes Apoptosis of NSCLC Cells

**Figure 1:**
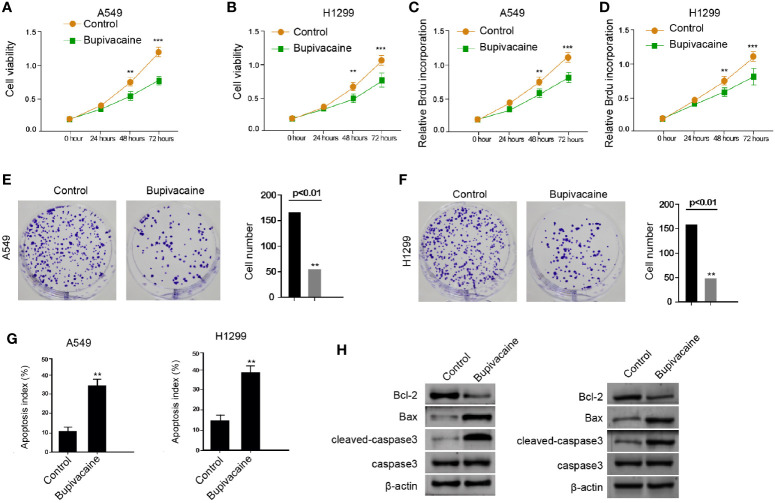
Bupivacaine inhibits proliferation and promotes apoptosis of NSCLC cells. **(A–H)** The A549 and H1299 cells were treated with bupivacaine (1 mM) or equal volume saline. **(A, B)** The cell viability was analyzed by the MTT assays in the cells. **(C, D)** The cell proliferation was tested by BrdU incorporation assays in the cells. **(E, F)** The cell proliferation was measured by the colony formation assays in the cells. **(G)** The cell apoptosis was measured by flow cytometry analysis in the cells. **(H)** The expression of Bcl-2, Bax, and cleaved caspase3 was determined by Western blot analysis in the cells. Data are presented as mean ± SD. Statistic significant differences were indicated: **P < 0.01, ***P < 0.001.

To assess the potential function of bupivacaine in the modulation of NSCLC progression, the human NSCLC A549 and H1299 cells were treated with bupivacaine. MTT assays showed that the treatment of bupivacaine significantly reduced the cell viability of the A549 and H1299 cells (**Figures 1A, B**). Meanwhile, BrdU incorporation assays showed that bupivacaine reduced the proliferation of A549 and H1299 cells (**Figures 1C, D**). Similarly, the colony formation was decreased by the bupivacaine treatment in the A549 and H1299 cells (**Figures 1E, F**). Moreover, cell apoptosis was induced by the treatment of bupivacaine in the cells (**Figure 1G**). Consistently, the Bcl-2 expression was decreased and the Bax and cleaved caspase3 expression were increased by bupivacaine in the cells (**Figure 1H**). Together these data suggest that bupivacaine can inhibit proliferation and promote apoptosis of NSCLC cells.

### Bupivacaine Represses Invasion and Migration of NSCLC cells

**Figure 2:**
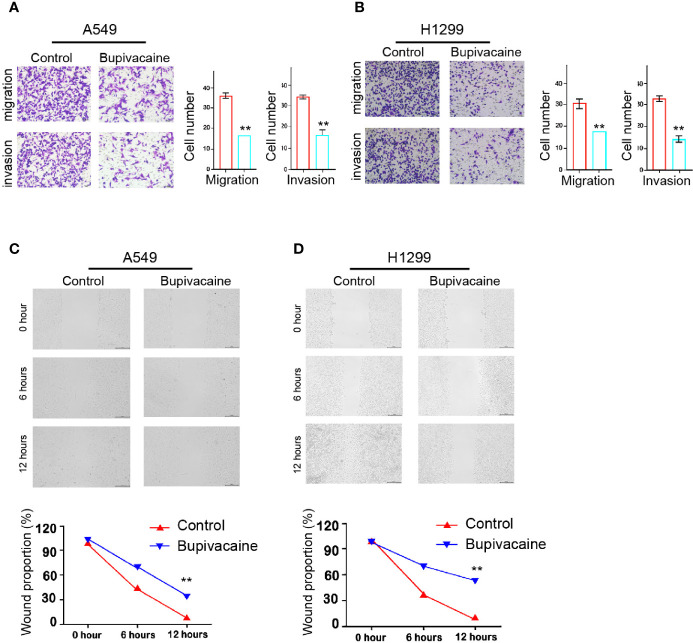
Bupivacaine represses invasion and migration of NSCLC cells. **(A–D)** The A549 and H1299 cells were treated with bupivacaine (1 mM) or equal volume saline. **(A, B)** The cell migration and invasion were examined by transwell assays in the cells. **(C, D)** The migration and invasion were measured by wound healing assays in the cells. The wound healing proportion was shown. Data are presented as mean ± SD. Statistic significant differences were indicated: **P < 0.01.

Then, we investigated the effect of bupivacaine on the migration and invasion of NSCLC cells. Transwell assays revealed that the migration and invasion of A549 and H1299 cells were remarkably inhibited by the treatment of bupivacaine (**Figures 2A, B**). Similarly, the treatment of bupivacaine remarkably enhanced the wound healing proportion in the cells (**Figures 2C, D**), suggesting that bupivacaine can decrease the migration and invasion of NSCLC cells *in vitro*.

### Bupivacaine Induces Autophagy in NSCLC Cells

**Figure 3:**
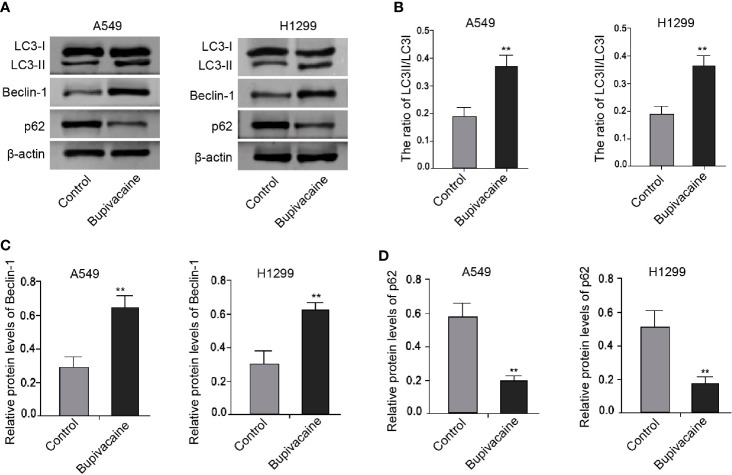
Bupivacaine induces autophagy in NSCLC cells. **(A–D)** The A549 and H1299 cells were treated with bupivacaine (1 mM) or equal volume saline. The expression of LC3B-II, LC3B-I, Beclin-1, p62, and β-actin was measured by Western blot analysis in the cells. The results of Western blot analysis were quantified by ImageJ software. Data are presented as mean ± SD. Statistic significant differences were indicated: **P < 0.01.

Next, we tried to explore the mechanism of bupivacaine-induced inhibitory impact on the NSCLC progression. Surprisingly, we identified that the treatment of bupivacaine enhanced the expression ratio of light chain 3B-II (LC3B-II)/LC3B-I and the expression of Beclin-1 in the A549 and H1299 cells (**Figures 3A–C**). Meanwhile, the expression of the autophagic adaptor protein p62 was reduced by bupivacaine treatment in the cells (**Figure 3D**). Together, these results suggest that bupivacaine is able to induce autophagy in the NSCLC cells.

### Bupivacaine Induces Autophagy by Inhibiting AKT/mTOR Signaling in NSCLC Cells

**Figure 4:**
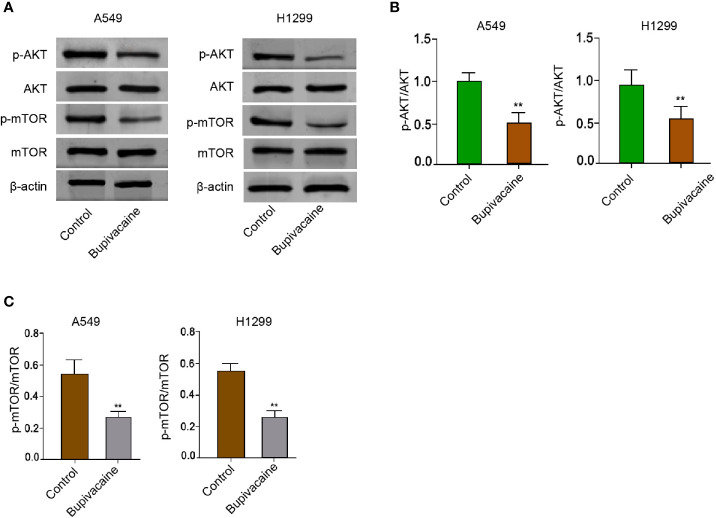
Bupivacaine induces autophagy by inhibiting AKT/mTOR signaling in NSCLC cells. **(A–C)** The A549 and H1299 cells were treated with bupivacaine (1 mM) or equal volume saline. The expression of AKT, mTOR, and β-actin, and the phosphorylation of AKT and mTOR was measured by Western blot analysis in the cells. The results of Western blot analysis were quantified by ImageJ software. Data are presented as mean ± SD. Statistic significant differences were indicated: **P < 0.01.

Given that Akt/mTOR signaling plays an essential role in the modulation of autophagy during NSCLC progression, we further explored whether bupivacaine induced autophagy by regulating AKT/mTOR signaling in the NSCLC cells. Significantly, the treatment of bupivacaine attenuated the phosphorylation of AKT and mTOR in the A549 and H1299 cells (**Figures 4A–C**). Together these data indicate that bupivacaine induces autophagy by inhibiting AKT/mTOR signaling in the NSCLC cells.

### Bupivacaine Attenuates NSCLC Progression by Inducing Autophagy Through Inhibiting AKT/mTOR Signaling *In Vitro*

**Figure 5:**
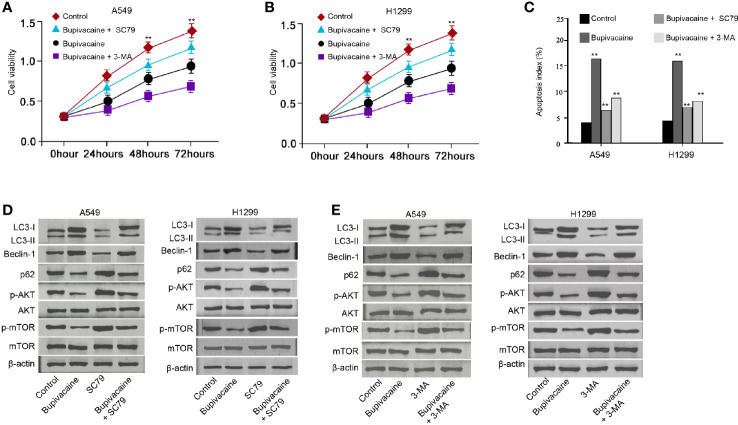
Bupivacaine attenuates NSCLC progression by inducing autophagy through inhibiting AKT/mTOR signaling *in vitro*. **(A–C)** The A549 and H1299 cells were treated with bupivacaine (1 mM) or equal volume saline, or cotreated with bupivacaine (1 mM) and SC79 (5 μM) or 3-MA (5 mM). **(A, B)** The cell viability was analyzed by the MTT assays in the cells. **(C)** The cell apoptosis was measured by flow cytometry analysis in the cells. **(D)** The A549 and H1299 cells were treated with bupivacaine (1 mM), SC79 (5 μM) or equal volume saline, or cotreated with bupivacaine (1 mM) and SC79 (5 μM). The expression of LC3B-II, LC3B-I, Beclin-1, p62, AKT, mTOR, and β-actin, and the phosphorylation of AKT and mTOR was tested by Western blot analysis in the cells. **(E)** The A549 and H1299 cells were treated with bupivacaine (1 mM), 3-MA (5 mM) or equal volume saline, or cotreated with bupivacaine (1 mM) and3-MA (5 mM). The expression of LC3B-II, LC3B-I, Beclin-1, p62, AKT, mTOR, and β-actin, and the phosphorylation of AKT and mTOR was tested by Western blot analysis in the cells. Data are presented as mean ± SD. Statistic significant differences were indicated: **P < 0.01.

We then explored the role of the bupivacaine/AKT/mTOR/autophagy axis in NSCLC development *in vitro*. MTT assays revealed that the bupivacaine treatment reduced the cell viability of A549 and H1299 cells, in which the autophagy inhibitor 3-methyladenine (3-MA) or AKT activator SC79 could rescue this reduction (**Figures 5A, B**). 3-MA or SC79 inhibited the cell apoptosis induced by the bupivacaine treatment in the cells (**Figure 5C**). Moreover, the AKT activator SC79 could rescue the bupivacaine-inhibited phosphorylation of AKT and mTOR in the cells (**Figure 5D**). Meanwhile, the treatment of bupivacaine increased the expression ratio of light chain 3B-II (LC3B-II)/LC3B-I and the expression of Beclin-1 in the A549 and H1299 cells, in which SC79 could reverse this phenotype (**Figure 5D**). In addition, SC79 was able to enhance the expression of p62 inhibited by bupivacaine in the system (**Figure 5D**). Similarly, 3-MA rescued the bupivacaine-inhibited phosphorylation of AKT and mTOR in the cells (**Figure 5E**). The treatment of bupivacaine enhanced the expression ratio of light chain 3B-II (LC3B-II)/LC3B-I and the expression of Beclin-1 in the A549 and H1299 cells, in which 3-MA could reverse this phenotype (**Figure 5E**). In addition, 3-MA could increase the expression of p62 inhibited by bupivacaine in the system (**Figure 5E**). Together these suggest that bupivacaine attenuates NSCLC progression by inducing autophagy through inhibiting Akt/mTOR signaling *in vitro.*

### Bupivacaine Inhibits the Tumor Growth of NSCLC *In Vivo*

**Figure 6:**
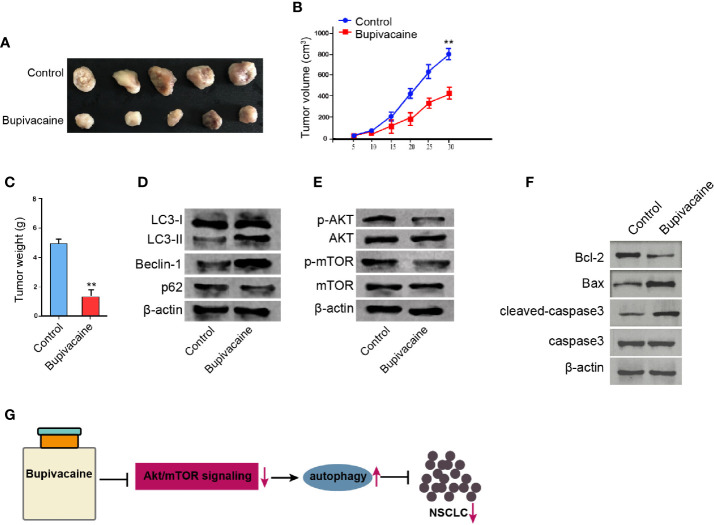
Bupivacaine inhibits the tumor growth of NSCLC *in vivo.* **(A–E)** The effect of bupivacaine on tumor growth of NSCLC cells *in vivo* was analyzed by nude mice tumorigenicity assay by injected with the A549 cells. The mice were treated with bupivacaine (40 μmol/kg) or equal volume saline. **(A)** Representative images of dissected tumors from nude mice were presented. **(B)** The average tumor volume was calculated and shown. **(C)** The average tumor weight was calculated and shown. **(D)** The expression of LC3B-II, LC3B-I, Beclin-1, p62, and β-actin was measured by Western blot analysis in the tumor tissues of the mice. **(E)** The expression of AKT, mTOR, and β-actin, and the phosphorylation of AKT and mTOR was assessed by Western blot analysis in the tumor tissues of the mice. **(F)** The expression of Bcl-2, Bax, and cleaved caspase3 was determined by Western blot analysis in the tumor tissues of the mice. **(G)** A model diagram of this investigation. Data are presented as mean ± SD. Statistic significant differences were indicated: **P < 0.01.

We further determined the impact of bupivacaine on the NSCLC development *in vivo*. For this purpose, we performed the tumorigenicity analysis in nude mice injected with A549 cells and were treated with bupivacaine. The treatment of bupivacaine significantly reduced the tumor growth of A549 cells *in vivo*, as demonstrated by the tumor size (**Figure 6A**), tumor volume (**Figure 6B**), and tumor weight (**Figure 6C**). Besides, the treatment of bupivacaine promoted the expression ratio of light chain 3B-II (LC3B-II)/LC3B-I and the expression of Beclin-1 in the tumor tissues (**Figure 6D**). The expression of p62 was repressed by bupivacaine treatment (**Figure 6D**). The treatment of bupivacaine also inhibited the phosphorylation of AKT and mTOR in the system (**Figure 6E**). Meanwhile, the Bcl-2 expression was decreased and the Bax and cleaved caspase3 expression were increased by bupivacaine in the tumor tissues (**Figure 6F**). Together these suggest that bupivacaine is able to inhibit the tumor growth of NSCLC *in vivo.*

## Discussion

NSCLC is the predominant type of lung cancer with severe morbidity and high mortality (18). The local anesthetic bupivacaine has presented potential anti-tumor activity in several cancer models (9, 10). Nevertheless, the role of bupivacaine in the development of NSCLC is still unreported. In this study, we firstly identified that bupivacaine could induce an inhibitory effect on the progression of NSCLC by activating autophagy through Akt/mTOR signaling.

As a widely used local anesthetic, bupivacaine has shown potential anti-cancer activity in several cancer types. It has been reported that bupivacaine inhibits gastric cancer development by multiple mechanisms independent of sodium channel blockade (19). Bupivacaine inhibits colon cancer cell proliferation *in vitro* (20). Bupivacaine induces an anti-cancer activity to pancreatic cancer *in vitro* (10). Bupivacaine presents anti-tumor properties by activating extrinsic and intrinsic apoptotic pathways in ovarian cancer and prostate cancer (9). Bupivacaine promotes apoptosis by caspase-independent and -dependent signaling in canine mammary tumor cells (21). Bupivacaine induces apoptosis by the mitogen-activated protein kinase pathway in human thyroid cancer cells (22). In this study, we identified that bupivacaine inhibited proliferation, invasion, and migration and induced apoptosis of NSCLC cells. Bupivacaine was able to attenuate the tumor growth of NSCLC *in vivo*. These data display a novel anti-tumor function of bupivacaine in NSCLC progression, providing valuable evidence for the fundamental role of the local anesthetic in the modulation of NSCLC.

As a primary cellular process, autophagy is widely involved in the development of NSCLC. It has been reported that Licarin A promotes cell death by activating apoptosis and autophagy of NSCLC cells (23). PDIA6 regulates autophagy and apoptosis by the MAP4K1-JNK signaling in NSCLC cells (24). MiR-384 induces autophagy and apoptosis by the negative modulation of the Collagen α-1(X) chain gene in NSCLC cells (25). Autophagy is accompanied by bisdemethoxycurcumin-related apoptosis in NSCLC cells (26). The inhibition of autophagy in cancer stem cells improves cisplatin’s efficacy for NSCLC (27). Furthermore, Akt/mTOR signaling significantly participates in the modulation of autophagy in NSCLC cells. It has been identified that 9za activates cytoprotective autophagy and induces the proapoptotic and cytotoxic effect by modulating the PDK1/Akt/mTOR signaling in NSCLC (28). Ganoderic acid DM provokes autophagic apoptosis by repressing the PI3K/Akt/mTOR signaling in NSCLC cells (29). Sotetsuflavone promotes autophagy in NSCLC cells by inhibiting PI3K/Akt/mTOR axis *in vitro* and *in vivo* (30). An active drug sensitizing agent improves gefitinib therapy by up-regulating autophagy and down-regulating PI3K/Akt/mTOR signaling in NSCLC (31). MiR-181 modulates cisplatin-resistant NSCLC by down-regulation of autophagy through the PI3K/AKT signaling (32). Our mechanical investigation further demonstrated that bupivacaine induced autophagy by inhibiting Akt/mTOR signaling in NSCLC cells and bupivacaine could attenuate NSCLC progression by inducing autophagy through inhibiting Akt/mTOR signaling. These data display an unreported correlation of bupivacaine with Akt/mTOR signaling-mediated autophagy in the development of NSCLC, identifying the new mechanism of bupivacaine-induced anti-tumor activity.

In conclusion, we discovered that the local anesthetic bupivacaine inhibited the progression of NSCLC by inducing autophagy through Akt/mTOR signaling (**Figure 6G**). Our finding provides new insights into the mechanism by which bupivacaine attenuates the development of NSCLC. Bupivacaine may serve as a potential anti-tumor candidate for the therapeutic strategy of NSCLC.

## Data Availability Statement

The original contributions presented in the study are included in the article/supplementary material. Further inquiries can be directed to the corresponding author.

## Author Contributions

J-HG and C-CL contributed to the conception or design of the work. J-LX and BM contributed to the acquisition, analysis, or interpretation of data for the work. S-MC and G-ZY drafted the manuscript. S-CH critically revised the manuscript. All authors contributed to the article and approved the submitted version.

## Conflict of Interest

The authors declare that the research was conducted in the absence of any commercial or financial relationships that could be construed as a potential conflict of interest.
